# Novel Role of Mammalian Cell Senescence-Sustenance of Muscle Larvae of *Trichinella* spp

**DOI:** 10.1155/2022/1799839

**Published:** 2022-11-28

**Authors:** Magdalena Dabrowska, Agnieszka Kępczyńska, Katarzyna Goździk, Małgorzata Całka-Kresa, Marek Skoneczny, Zbigniew Zieliński, Maria Doligalska, Ewa Sikora

**Affiliations:** ^1^Laboratory of Molecular Bases of Ageing, Nencki Institute of Experimental Biology, Polish Academy of Sciences, Warszawa 02-093, Poland; ^2^Laboratory of Electron Microscopy, Nencki Institute of Experimental Biology, Polish Academy of Sciences, Warszawa 02-093, Poland; ^3^Department of Parasitology, Institute of Functional Biology and Ecology, Faculty of Biology, University of Warsaw, Warszawa 02-089, Poland; ^4^Laboratory of Imaging Tissue Structure and Function, Nencki Institute of Experimental Biology, Polish Academy of Sciences, Warszawa 02-093, Poland; ^5^Laboratory of Yeast Genetics and Molecular Biology, Institute of Biochemistry and Biophysics, Polish Academy of Sciences, Warszawa 02-106, Poland; ^6^Laboratory of Molecular Bases of Cell Motility, Nencki Institute of Experimental Biology, Polish Academy of Sciences, Warszawa 02-093, Poland

## Abstract

Muscle larva of the parasitic nematode *Trichinella* spp. lives in a portion of muscle fibre transformed to a nurse cell (NC). Based on our previous transcriptomic studies, NC growth arrest was inferred to be accompanied by cellular senescence. In the current study, NC was proven to display the following markers of senescence: high senescence-associated *β*-galactosidase activity, lipid deposition, DNA damage, and cell cycle inhibition. Moreover, the nuclear localization of Activator Protein 1 (c-Fos, c-Jun, and FosB), as well as the upregulation of numerous AP-1 target genes in the NC, remained in accord with AP-1 recently identified as a master transcription factor in senescence. An increase in reactive oxygen species generation and the upregulation of antioxidant defence enzymes, including glutathione peroxidases 1 and 3, catalase, superoxide dismutases 1 and 3, and heme oxygenase 1, indicated an ongoing oxidative stress to proceed in the NC. Interestingly, antioxidant defence enzymes localized not only to the NC but also to the larva. These results allowed us to hypothesize that oxidative stress accompanying muscle regeneration and larval antigenic properties lead to the transformation of a regenerating myofibre into a senescent cell. Cellular senescence apparently represents a state of metabolism that sustains the long-term existence of muscle larva and ultimately provides it with the antioxidant capacity needed during the next host colonization. Senotherapy, a therapeutic approach aimed at selective elimination of senescent cells, can thus be viewed as potentially effective in the treatment of trichinosis.

## 1. Introduction

Cellular senescence is a metabolically active state of stable growth arrest which occurs in response to various types of stress [1]. In normal dividing cells, replicative senescence is triggered due to telomere attrition. Exogenous stressful stimuli may lead both normal and cancer cells to accelerated senescence, also known as stress-induced premature senescence or therapy-induced senescence, respectively [2, 3]. Hypermitogenic stimulation that takes place during oncogenic transformation may cause oncogene-induced senescence which is considered a barrier to tumorigenesis [4]. Despite a variety of inducing factors, cellular senescence is characterized by a set of common morphological and molecular markers, with senescence-associated *β*-galactosidase (SA-*β*-gal) activity being the main hallmark [5]. In *in vivo* settings, cellular senescence is associated with the onset of organismal ageing and age-related disorders [6].

Muscle larvae of the parasitic nematode *Trichinella* spp. are the infective stage of the parasite. They develop in mammalian striated muscles from newborn larvae which after being delivered by adult female parasites in the small intestine, migrate with the lymph and blood to reach the muscles. Muscle larva develop and ultimately reside within the muscle tissue in a unique type of cell called a nurse cell (NC) [7]. A single NC usually accommodates one larva, although a few larvae were also seen (Supplementary Figure 1). The NC is formed from misdifferentiated muscle satellite cells which have fused with a parasite-invaded degenerating myofibre. Though originating from muscle cells, the NC is of the nonmuscular type. The molecular mechanism of NC development remains largely unknown. The basic structure of the NC, which ultimately becomes confined within a collagen capsule surrounded by a circulatory rete, develops within 20-28 days after ingestion of meat infected with the parasite muscle larvae [8]. The NC-larva complex is stably maintained throughout the life span of a host. It was found to last for up to 40 years in humans [9]. The NC is multinuclear and hypertrophic [10, 11]. Based on transcriptomic studies, its growth arrest was inferred to be of the G_1_-like type accompanied by cellular senescence. NC-specific signalling pathways and biological functions were delineated from putative autocrine/paracrine signalling loops. Hypermitogenic stimulation counterbalanced by cyclin-dependent kinase (CDK) inhibitor expression, as well as acquisition of immunological properties, underpinned by protein kinase C (PKC), mitogen-activated protein kinases p38 MAPK *α*/*δ*, Jun N-terminal kinase JNK3, and activator protein 1 AP-1-dependent signalling, were identified as the main determinants of NC functionality [12–14]. AP-1 is known to induce the expression of the subunits of the transmembrane NADPH oxidase complex, whose upregulation was also found to occur in the NC [13, 15]. Reactive oxygen species (ROS) generated by NADPH oxidase could serve signal transduction; however overproduction may exceed the cellular capacity of antioxidant defence and exert detrimental effects on macromolecules [16, 17]. Oxidative stress, though not named a marker of cellular senescence, was claimed to be a condition necessary for both senescence induction and maintenance [18, 19]. Oxidative stress is known to induce PKC, p38 MAPK, and JNK and ultimately to lead to the activation of the AP-1 transcription factor [20–23].

AP-1 has recently been ascribed a central role in the regulation of the transcriptional program of cellular senescence. It was shown to determine the pattern of active enhancers and the interactions within the network of active transcription factors [24]. Binding to enhancers rather than to proximal gene promoters is considered the main level of AP-1-dependent regulation of cellular senescence [25, 26]. AP-1 acts as a dimer composed of interchangeable subunits, with those belonging to the Jun and Fos subfamilies considered the prototypical AP-1 factors. The involvement of AP-1 in the regulation of various cellular functions, including the immune response, is attributed to the diversity of dimer combinations, their interactions with other transcription factors and binding not only to the consensus motifs but also to the related sequences [27, 28].

Since oxidative stress is viewed as a prerequisite for senescence establishment and AP-1 is one of the main cellular mediators of redox-sensitive signalling, the involvement of oxidative stress, antioxidant defence enzymes, and AP-1, including c-Fos, c-Jun, and FosB factors, in the maintenance of NC senescence was followed in the current study.

## 2. Materials and Methods

### 2.1. Trichinellosis

This was maintained in BALB/c mice infected with *Trichinella spiralis* reference isolate ISS1 obtained from the International *Trichinella* Reference Centre, Instituto Superiore di Sanita, Rome, Italy. Mice were infected *per os* with 200 L1 infective muscle larvae, as previously described [29]. Animals were kept in the Animal House Unit of University of Warsaw. The procedures were approved by the First Warsaw Local Ethics Committee for Animal Experimentation and carried out according to the Polish Law on Animal Experimentation and EU Directive 2010/63/UE.

### 2.2. NC Lysate Preparation

NCs were isolated from 5- to 12- month-old infections, as previously described [12]. Briefly, minced mouse carcasses were digested with 0.25% pronase, 0.1% collagenase, and 0.1% hyaluronidase, and the NC-larva complexes were recovered by centrifugation through a two-step Percoll gradient. The complexes from a typical preparation are shown in Supplementary Figure 2. The NCs were disrupted in lysis buffer from an RNeasy Mini kit (Qiagen, Hilden, Germany), and the NC lysate was recovered as a supernatant in a brief centrifugation in a microcentrifuge. This procedure left the larvae intact and collected in a pallet.

### 2.3. Tissue Processing

Skeletal muscles, tongue, and diaphragm were isolated from 7-month-old infections. The tissues were rinsed with phosphate buffered saline (PBS). The diaphragm was cut into slices that were either directly used in biochemical assays or were processed further for microscopic assays. The tissues were fixed with 4% formaldehyde (Sigma-Aldrich, Saint Louis, MO, USA), transferred through a three-step sucrose gradient (10, 20, and 30%), frozen on dry ice in Optimal Cutting Temperature compound (ThermoFisher Scientific, Waltham, MA, USA), and cut into 10 *μ*m sections on a Leica 1950 cryostat (Leica Microsystems, Wetzlar, Germany).

### 2.4. Transcriptomic Analyses

RT^2^ Profiler PCR array: Mouse Oxidative Stress and Antioxidant Defence was used according to the manufacturer's protocol (Qiagen). Briefly, total RNA was isolated from the NC lysate using an RNeasy Mini kit with the implementation of an RNase-Free DNase digestion step. RNA quality was confirmed using an Agilent 2100 BioAnalyzer (Agilent, Santa Clara, CA, USA). Reverse transcription was performed using an RT^2^ First Strand kit. Arrays were run on an Applied Biosystems 7500 Sequence Detection System (ThermoFisher Scientific) using RT^2^ SYBR Green qPCR Master Mix. The target gene expression level was calculated by applying the comparative threshold cycle (C_T_) method, with glyceraldehyde-3-phosphate dehydrogenase (Gapdh) used as a reference gene. It is given as 2exp − ΔC_T_ (±AD, *N* = 2), where *Δ*C_T_ is C_T_ (target)–C_T_ (Gapdh). Competitive expression microarray datasets profiling the NC transcriptome in referrence to the transcriptomes of murine C2C12 myoblasts and myotubes, available in ArrayExpress database at https://www.ebi.ac.uk/arrayexpress, under the accession E-MTAB-12042, were exploited for three types of analyses. They were searched for changes in the expression level of (i) genes involved in oxidative stress and antioxidant defence and (ii) AP-1 target genes and were also employed for (iii) global functional analysis. Only genes with expression parameters −0.5 ≥ log_2_FoldChange (FC) ≥ 0.5 and *p* − value ≤ 0.05 were included in each analysis. The gene list available at http://rulai.cshl.edu/TRED/GRN/AP1.htm was used as the main source of canonical AP-1 target genes. Functional analysis of datasets was performed in Qiagen Ingenuity Pathway Analysis software [30]. The heatmaps were created in Morpheus software available at https://software.broadinstitute.org/morpheus.

### 2.5. SA-*β*-Gal Activity Assay

SA-*β*-gal activity was assessed in freshly isolated diaphragm by a colorimetric method [31]. Slices of the diaphragm were fixed with 2% formaldehyde, 0.2% glutaraldehyde PBS solution, and then exposed to a solution containing 1 mg/ml 5-bromo-4-chloro-3-indolyl-*β*-D-galactopyranoside (ThermoFisher Scientific), 5 mM potassium ferrocyanide, 5 mM potassium ferricyanide, 150 mM NaCl, 2 mM MgCl_2_, and 0.1 M phosphate buffer pH 6.0 for 4 h at 37°C. Images were taken with a Leica DMI 6000 microscope (Leica Microsystems).

### 2.6. ROS Measurement

Slices of freshly isolated diaphragm were fixed with 3.7% formaldehyde PBS solution and incubated with 5 *μ*M CellROX Green Oxidative Stress Reagent and 1 *μ*g/ml Hoechst 33342 PBS solution (both from ThermoFisher Scientific) for 30 min at 37°C. The slices were imaged with an Axio Observer Z.1 inverted microscope (Carl Zeiss, Jena, Germany) equipped with CSU-X1 spinning disc unit (Yokogawa, Tokyo, Japan), Evolve 512 EMCCD camera (Teledyne Photometrics, Tucson, AZ, USA), diode 405/488/561/639 nm lasers and 20x0.8NA objective (Carl Zeiss). Z-stacks were collected using ZEN software (Carl Zeiss). Quantitative analysis of CellROX Green fluorescence was performed in Imaris 9.1.2 software (Andor Technology, Belfast, UK). The results are presented as CellROX Green fluorescence intensity in the nuclei normalized to the nuclear area measured after Hoechst 33342 labelling.

### 2.7. Oil Red O (ORO) Labelling

Tissue sections immobilized on microscopic slides were stained with ORO solution, counterstained with haematoxylin Mayer's solution (both from Sigma-Aldrich), and mounted using Faramount Mounting Medium, Aqueous (Dako, Agilent). All chemicals were applied according to the manufacturers' protocols. The images were taken using a Nikon Eclipse 50i microscope (Nikon Instruments, Tokyo, Japan).

### 2.8. Immunohistochemistry

Tissue sections immobilized on microscopic slides were allowed to equilibrate to room temperature, rehydrated in PBS, exposed to Hydrogen Peroxide Blocking Reagent (Abcam, Cambridge, UK), blocked with 5% bovine serum albumin (Sigma-Aldrich), 5% either goat or donkey serum (both from ThermoFisher Scientific), and 0.5% Triton X-100 PBS solution, and incubated with primary antibodies overnight at 4°C. The following set of primary antibodies was used: glutathione peroxidase GPX1/2 and superoxide dismutase SOD1, both gifts from the Laboratory of Cellular Metabolism at Nencki Institute, coming from Santa Cruz Biotechnology, Dallas, TX, USA as H-151 and FL-154, respectively, superoxide dismutase SOD2 (D3X8F) from Cell Signaling Technology, Danvers, MA, USA, glutathione peroxidase GPX3 (AF4199-SP) from R&D Systems (Bio-Techne, Minneapolis, MN, USA), catalase (NBP2-24916SS) and superoxide dismutase SOD3 (NBP1-46612) from Novus (Bio-Techne). The specificity of the immune recognition of proteins within *T. spiralis* larvae was confirmed using the second set of primary antibodies: GPX1 (C5-A10), SOD1 (PA5-85095), SOD2 (PA5-31072), SOD3 (PA5-102904), catalase (PA5-29183), and GPX3 (PA5-18677) from Invitrogen (ThermoFisher Scientific). The results obtained with the first set of the antibodies are presented. Horse radish peroxidase-conjugated donkey anti-goat IgG (Santa Cruz Biotechnology) or Dako goat anti-rabbit IgG (Agilent) were used as secondary antibodies. The samples were labelled using a 3,3′ diaminobenzidine (DAB) Substrate kit (Abcam), counterstained with haematoxylin Meyer's solution, dehydrated in a three-step ethyl alcohol gradient and xylene, and mounted using Limonene Mounting Medium (Abcam). The images were collected using an Olympus VS110 microscope (Olympus, Tokyo, Japan).

### 2.9. Immunofluorescence

Tissue sections immobilized on microscopic slides were allowed to equilibrate to room temperature, rehydrated in PBS, blocked with 5% bovine serum albumin, 5% either goat or donkey serum and 0.5% Triton X-100 PBS solution, and incubated with primary antibodies overnight at 4°C. The primary antibodies used were those from the first set of the antibodies applied in the immunohistochemistry, as well as p57 Kip2 [EP2515Y] and *γ*-H2A.X (phospho S139) [9F3] from Abcam, 53 Binding Protein 1 (NB100-304) from Novus (Bio-Techne), heme oxygenase HO-1 (ADI-OSA-150) from Enzo Life Sciences, Farmingdale, New York, NY, USA, c-Jun (60A8), phospho-c-Fos (Ser32) (D82C12), and FosB (5G4) from Cell Signaling Technology. Invitrogen goat anti-rabbit IgG (H+L) and donkey anti-goat IgG (H+L), both Alexa Fluor 488 conjugates (ThermoFisher Scientific), were used as secondary antibodies. The nuclei were labelled with Hoechst 33342 1 *μ*g/ml PBS solution. The tissue sections were mounted using Invitrogen Fluoromount-G Mounting Medium (ThermoFisher Scientific). The images were collected with a Zeiss Cell Discoverer 7 + LSM 900 confocal microscope equipped with Plan-Apochromat 20x/0.95 DRY, Optovar 2x Tubelens lens (Carl Zeiss). The whole NC-larva complexes were imaged at a pinhole size of 67 *μ*m (0.90 AU for Alexa Fluor 488), 0.5 *μ*m optical section interval, and 104 nm x 104 nm x 500 nm voxel size. Ex/Em were set at 493 nm/493-581 nm for Alexa Fluor 488 and 405 nm/400-476 nm for Hoechst 33342. High-resolution images of larval stichosome areas were collected with a high sampling method in AiryScan mode. The optical section interval was 0.190 *μ*m, and the voxel size was 69 nm x 69 nm x 190 nm. Ex/Em were set at 493 nm/450-700 nm for Alexa Fluor 488 and 405 nm/400-605 nm for Hoechst 33342. Z-stacks were collected in ZEN 3.4 Blue software (Carl Zeiss). SuperResolution Mode (3D, Auto) was used for AiryScan processing. Quantitative analysis of HO-1 expression levels was performed in Fiji software (http://imagej.net/Fiji). The results are presented as Alexa Fluor 488 fluorescence intensity normalized to the cell area.

### 2.10. Statistics

The significance of the differences between the groups was assessed using a nonparametric Kruskal-Wallis test performed in Statistica 13.3 software (TIBCO, Palo Alto, CA, USA). A probability value of *P* ≤ 0.05 was considered significant.

## 3. Results

### 3.1. NC Senescence Is Underpinned by Oxidative Stress, DNA Damage and p57 Kip2 Expression

**Figure 1:**
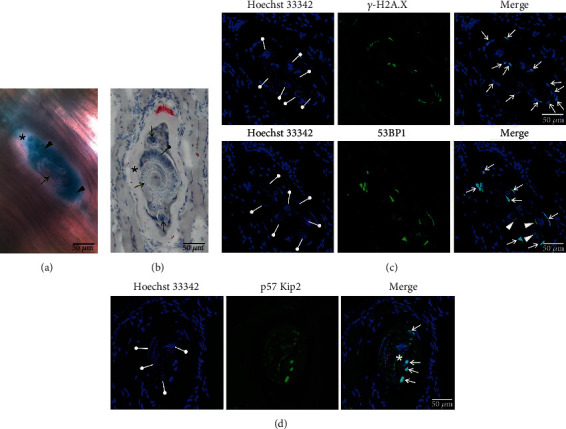
Senescence markers in the NC. (a) SA-*β*-gal activity evident by blue staining of the NC cytoplasm in a slice of diaphragm. (b) Lipid droplet deposition at the apex of the NC capsule visualized by Oil Red O staining in a diaphragm section. In (a) and (b), black asterisks indicate collagen capsules and black arrows indicate the larvae. Black arrowheads in (a) point to the NC cytoplasm, and black drumstick in (b) points to the larval stichosome. (c) and (d) *γ*-H2A.X, 53BP1, and p57 Kip2 localization in the NC assayed by immunofluorescence in the muscle sections. In Hoechst 33342 images, white drumsticks indicate larval nuclei. In the merged images, white arrows indicate NC nuclei, white arrowheads indicate 53BP1 aggregates in the larva, and white asterisk points to the cuticle and subcuticular muscle layer of the larva.

**Figure 2:**
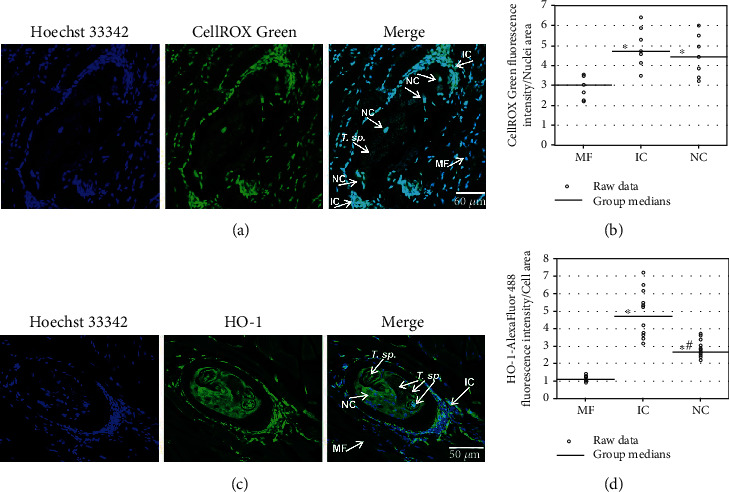
Oxidative stress manifestations in the NC. (a, b) Reactive oxygen species level depicted by CellROX Green fluorescence intensity in the nuclei of various cell populations in a slice of diaphragm. (c, d) Heme oxygenase HO-1 levels in muscle sections assayed by immunofluorescence. MF indicates muscle fibre nuclei in (a) or muscle fibre areas in (c), IC indicates infiltrating cell nuclei in (a) or infiltrating cell clusters in (c), NC indicates NC nuclei in (a) or NC cytoplasm in (c), *T.* sp. indicates larval nuclei in (a) or larva sections in (c). ^∗^, *p* − value < 0.04 in (b) and < 0.00 in (d) *vs.* MF for *N* = 7 in (b) and *N* ≥ 12 in (d); ^#^, *p* − value < 0.03 *vs.* IC for *N* ≥ 12.

**Figure 3:**
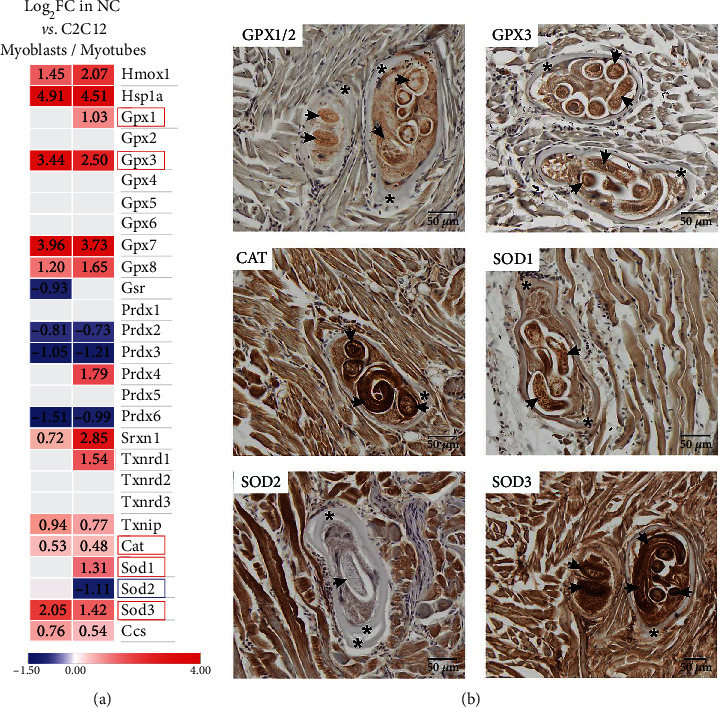
Expression of antioxidant defence enzymes in the NC-larva complex. (a) Heatmap shows gene expression in the NC in reference to C2C12 myoblasts and myotubes, as analyzed by microarrays. The lack of a figure in the column indicates unchanged gene expression. Boxes indicate the genes analyzed by immunohistochemistry in (b). (b) Expression of selected enzymes in the muscle sections analyzed by immunohistochemistry. Arrows point to representative larva sections within the NC and asterisks point to representative fields of the collagen capsule. Cat, catalase; Ccs, copper chaperone for SOD1; Gpx, glutathione peroxidase; Gsr, glutathione reductase; Hmox1, heme oxygenase; Prdx, peroxiredoxin; Sod, superoxide dismutase; Srxn1, sulfiredoxin; Txnrd, thioredoxin reductase; Txnip, thioredoxin interacting protein.

High SA-*β*-gal activity was detected in both NC preparations, a freshly isolated diaphragm (Figure 1(a)) and an individual NC obtained from muscles by enzymatic digestion (Supplementary Figure 3). Additionally lipid deposition, which is a facultative senescence marker allowing the visualization of senescent cells within tissues, was found to occur at the apex of a collagen capsule surrounding the NC (Figure 1(b)). In comparison to the muscle fibres, ROS production was increased in the NC, as well as in the infiltrating cells forming clusters around it (Figures 2(a) and 2(b)). This was paralleled by the upregulation of the oxidative stress marker heme oxygenase 1, at both the transcript (Hmox1) and protein levels (HO-1) (Figure 2(c) and Figure 3(a)). The median HO-1 expression in the NC despite being 2.4-fold higher than that in the muscle fibres, was 1.8-fold lower than that in the infiltrating cells (Figure 2(d)). It should be noted that weak CellROX Green fluorescence indicating intracellular ROS levels, as well as HO-1, was also detected in the larva. Two markers of DNA damage, a phosphorylated form of histone H2A, *γ*-H2A.X, and 53BP1, were detected in the NC nuclei (Figure 1(c)). A weak signal from 53BP1 was also found in the larval body, however, outside of the nuclei. Finally, cell cycle arrest of the NC was documented by CDK inhibitor p57 Kip2 upregulation (Figure 1(d)). p57 Kip2 localized mainly to the NC nuclei and cytoplasm. It was also detected at the larval cuticle region (cuticle and muscle layer beneath the cuticle) but not in the larval nuclei.

### 3.2. Antioxidant Defence Enzymes Are Upregulated in the NC-Larva Complex

Transcriptomic analyses revealed the upregulation of several antioxidant defence enzymes in the NC (Figure 3(a) and Supplementary Table 1). The glutathione peroxidases Gpx1, Gpx3, Gpx7, and Gpx8 were upregulated in comparison to those in C2C12 myoblasts and myotubes. Glutathione reductase Gsr expression was maintained in the NC at the same level as in C2C12 myotubes. Peroxiredoxins' (Prdx) expression was vastly downregulated. Only Prdx4 was upregulated in comparison C2C12 myotubes. Sulfiredoxin Srxn1 was upregulated in comparison to that in C2C12 myoblasts and myotubes. Thioredoxin reductase Txnrd1 was upregulated in the NC in relation to C2C12 myotubes only; however, the thioredoxin inhibiting factor-coding gene Txnip was also upregulated. The basic ROS neutralizing enzyme-coding genes, including catalase Cat, superoxide dismutase Sod1 and Sod3, but not Sod2, were upregulated. Additionally, a copper chaperone for SOD1, Ccs, was upregulated. The GPX1, GPX3, catalase, SOD1, SOD2, and SOD3 expression patterns in the NC were confirmed at the protein level (Figure 3(b)). The enzymes were detected not only in the NC but also in the larva, with SOD1 expression being higher in the larva only but not in the NC when compared to the surrounding muscle tissue. SOD2 expression was confirmed to be higher in the muscles than in the NC-larva complex. This expression pattern was obtained using two different sets of antibodies, as described in the Materials and Methods.

### 3.3. Antioxidant Defence Enzymes Localize to the NC Cytoplasm, as well as to the Stichosome and Cuticular Region of the Larva

**Figure 4:**
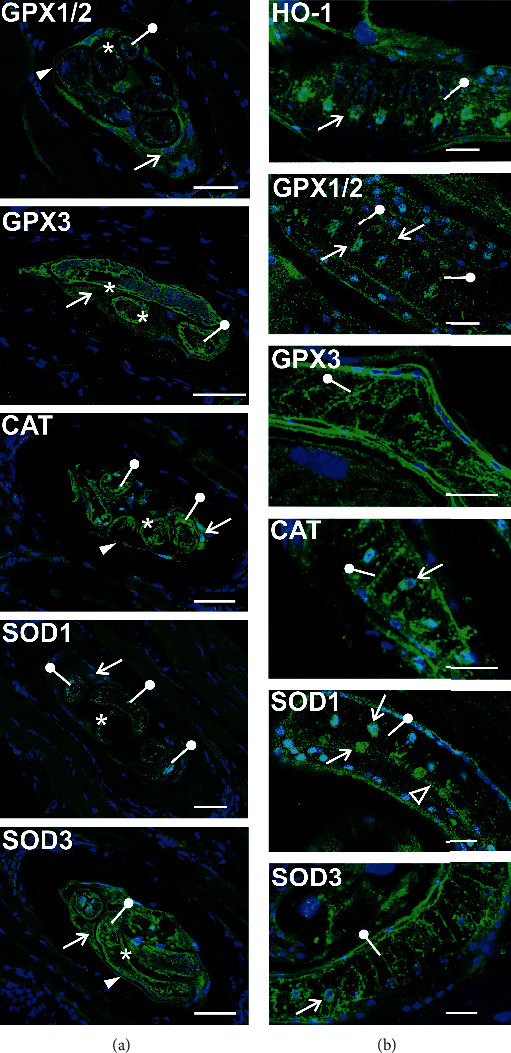
Localization of selected antioxidant defence enzymes in the NC-larva complex analyzed by immunofluorescence in the muscle sections. (a) Arrows point to the NC cytoplasm, arrowheads indicate the NC plasma membrane, asterisks indicate larval cuticular regions, and drumsticks indicate larval internal organs. Scale bar, 50 *μ*m. (b) The images show fragments of larval stichosome. Arrows point to the stichocyte nuclei, drumsticks indicate the stichocyte canaliculi, and empty arrowhead points to the esophagus wall. Scale bar, 10 *μ*m. CAT, catalase; GPX, glutathione peroxidase; HO-1, heme oxygenase; SOD, superoxide dismutase.

Localization of the antioxidant enzymes in the NC-larva complex was analyzed by confocal microscopy (Figure 4(a)). All enzymes except SOD1 were detected in the NC cytoplasm. GPX1, catalase, and SOD3 were also located in the plasma membrane region of the NC. All enzymes were also present in the larval cuticle, muscle layer beneath the cuticle, and larval internal organs. Specifically, the enzymes were found in the stichosome, which is an excretory-secretory organ of the parasite. It surrounds the esophagus and is built from cylindrical cells whose nuclei form rows alongside the esophagus [32]. As shown in Figure 4(b), all antioxidant defence enzymes analyzed were found in the canaliculi of stichocytes. GPX1, catalase, and SOD1 were also located in the stichocyte nuclei, and SOD1 was also found lining the esophagus wall. HO-1, which was upregulated in the NC-larva complex (Figure 2(c)), was also present in the stichocyte canaliculi and nuclei (Figure 4(b)).

### 3.4. AP-1: c-Fos, c-Jun, and FosB Expression Is Induced in the NC

**Figure 5:**
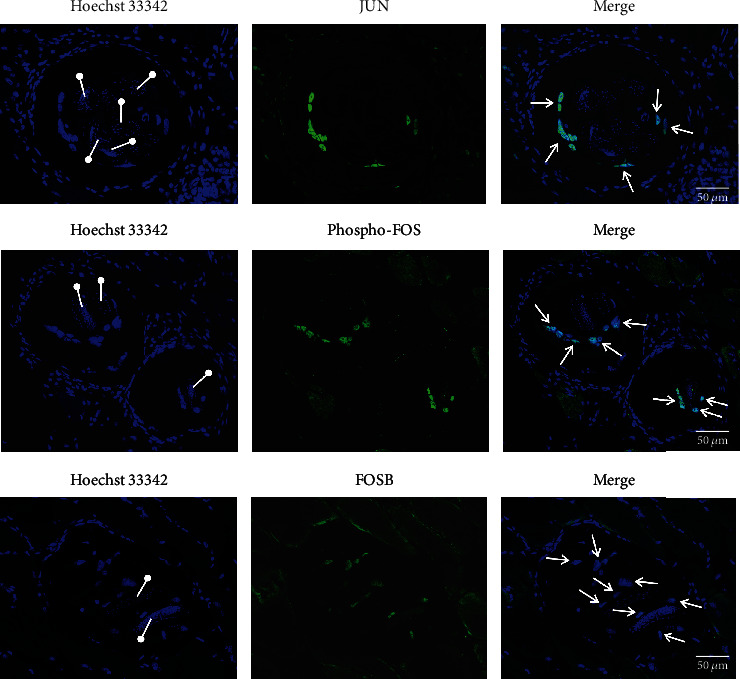
AP-1: JUN, Phospho-FOS and FOSB expression in the NC nuclei analyzed by immunofluorescence in the muscle sections. Arrows point to the NC nuclei in merge images, whereas drumsticks point to the clusters of the parasite nuclei in Hoechst 33342 images.

**Figure 6:**
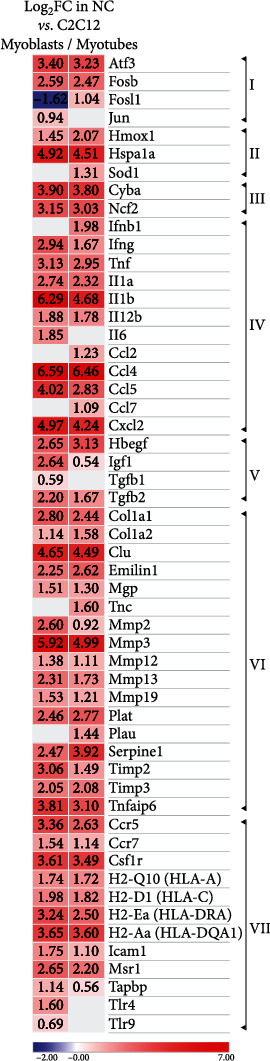
AP-1 target genes whose expression was induced in the NC in comparison to C2C12 myoblasts and myotubes, as analyzed by microarrays. Genes were identified as AP-1 targets by referring microarray datasets to the canonical AP-1 target gene lists available at http://rulai.cshl.edu/TRED/GRN/AP1.htm, as well as in the references [15, 76-80]. The heatmap shows selected genes grouped into the following functional categories: I, AP-1 factors; II, oxidative stress-related factors; III, NADPH oxidase complex subunits; IV, cytokines and chemokines; V, growth factors; VI, extracellular matrix components and modulating factors; VII, immune response-related factors. The lack of a figure in the column indicates that gene expression did not change. A complete list of AP-1 target genes is presented in Supplementary Table 2. Gene symbols are explained in the Results section 3.5 and Supplementary Table 2.

The upregulation of c-Fos and FosB in the NC, as inferred from microarray analysis [12], was confirmed in this study to occur at the protein level (Figure 5). Since AP-1 factors are activated by phosphorylation, the presence in the NC nuclei of a phosphorylated form of Fos confirmed the factor activation. The AP-1 Jun component c-Jun was upregulated by 2-fold at the transcript level in relation to C2C12 myoblasts only (Figure 6), and was also confirmed to be upregulated in the NC at the protein level. FOS, JUN and FOSB localized solely to the nuclei of the NC.

### 3.5. AP-1 Effector Genes Are Upregulated in the NC

Functional analysis of expression microarray datasets performed in IPA software returned the AP-1 transcription factor as the upstream regulator of NC biological functions. The consistency scores with the QIAGEN Knowledge Base were 3.93 and 3.00 for the datasets referring the NC to C2C12 myoblasts and myotubes, respectively. The analysis also showed FosB as the top upregulated transcription factor determining causal regulatory networks of the NC, with *P* values 6.63E-53 and 7.63E-53 for the datasets referring the NC to C2C12 myoblasts and myotubes, respectively. Accordingly, the expression of numerous AP-1 effector genes was induced in the NC. A complete gene list is shown in Supplementary Table 2, whereas selected genes are presented in the heatmap (Figure 6). These genes belong to various functional categories. AP-1 factors, including activating transcription factor 3 (Atf3), Jun, Fosb, and Fos-like antigen 1 (Fosl1), are AP-1 targets themselves. Apart from Hmox1, the heat shock protein Hspa1a and Sod1 are also oxidative stress-associated AP-1 target genes. Two NADPH oxidase subunits, cytochrome b-245 alpha (Cyba), and neutrophil cytosolic factor 2 (Ncf2), are AP-1 targets. Interferons Ifnb1 and Ifng, tumor necrosis factor (Tnf), interleukins Il1a, Il1b, Il12b, Il6, chemokines Ccl2/4/5/7, and Cxcl2 are AP-1 target factors that apparently constitute the senescence-associated secretory phenotype (SASP), a feature considered a senescence marker [5]. Other AP-1 targets in the SASP of the NC were growth factors: heparin-binding epidermal growth factor-like growth factor (Hbegf), insulin-like growth factor 1 (Igf1), and transforming growth factors Tgfb1 and Tgfb2. The SASP is also known to contain extracellular matrix components and modulating factors. The NC SASP contained the following AP-1 target genes belonging to this category: collagens Col1a1 and Col1a2, clusterin (Clu), elastin microfibril interfacer 1 (Emilin1), tenascin C (Tnc), matrix Gla protein (Mgp), matrix metallopeptidases Mmp2/3/12/13/19, plasminogen activators of tissue (Plat), and urokinase (Plau) types, serine peptidase inhibitor Serpine1, tissue inhibitors of matrix metalloproteinases Timp2 and Timp3, and TNF-induced protein 6 (Tnfaip6). Chemokine receptors Ccr5 and Ccr7, colony stimulating factor 1 receptor (Csf1r), macrophage scavenger receptor 1 (Msr1), Toll-like receptors Tlr4, and Tlr9, intercellular adhesion molecule Icam1, major histocompatibility complex MHC class I molecules H2-Q10, and H2-D1 (their human counterparts are human leukocyte antigen HLA-A and HLA-C, respectively), MHC class II molecules H2-Ea and H2-Aa (their human counterparts are HLA-DRA and HLA-DQA1, respectively), and transporter associated with antigen processing binding protein (Tapbp) are all AP-1 target genes involved in the immune response whose expression was induced in the NC.

## 4. Discussion

Signalling pathway-directed analysis of microarray data led us previously to hypothesize that the stage of cell cycle arrest of mammalian cell accommodating *Trichinella* spp. muscle larva, an NC, was of the G_1_-like type accompanied by cellular senescence [12]. In the current study, the senescent state of a fully developed NC was functionally characterized. The main hallmark of cellular senescence, high SA-*β*-gal activity, was shown to occur in the NC. Elevated ROS levels pointed to oxidative stress as a cause of cellular damage leading to the establishment of NC senescence. Nuclear localization of an early DNA double strand break repair protein, *γ*-H2A.X, indicated that ongoing DNA damage proceeded in the NC. Additionally, 53BP1, the other DNA damage indicator considered a marker in senescence [33], was elevated in the NC nuclei. This remains in accord with the reasoning that ROS production leading to perpetual DNA damage is a precondition for senescence establishment, which depends on p38 MAPK activation [18]. Accordingly, p38 MAPK *α*/*δ* upregulation in NC was also found to occur [13]. A notion that oxidative stress shapes NC functionality was previously proposed by Bruschi [34]. Growth arrest of the NC was underpinned by p57 Kip2 CDK inhibitor expression upregulated at the transcript [12] and protein levels. Other CDK inhibitors, p16-INK4a, and p21^Waf1/Cip1^, are considered standard markers of cell cycle arrest in senescence [5]. However, due to the transient expression in muscle satellite cells and myonuclei [35], p16-INK4a is not considered suitable for studies of senescence in muscle tissue and as such was not tested. p21^Waf1/Cip1^ protein in turn was not found to be upregulated in the NC (not shown). When exerting an inhibitory role in the mammalian cell cycle, p57 Kip2 is predominantly located in cell nuclei [36], and it showed such a location in the NC. p57 Kip2 was also detected in the larval cuticle and muscle layer. This allows us to hypothesize that larva takes up the protein produced in the NC to support growth arrest of its own cells. Similarly, the oxidative stress marker HO-1 [37], which was upregulated in the NC at both the transcript and protein levels, was also detected in the larval internal organs. HO-1 may thus be used by the larva to combat ROS while in the NC or when infecting the next host. Oxidative stress induces the synthesis of antioxidant defence enzymes to neutralize ROS and protect or repair cellular macromolecules [20]. The expression pattern of antioxidant defence enzymes revealed that the major cellular protein repair system, which is dependent on thioredoxin, exerts a rather limited role in the NC. Thioredoxin is a physiological thiol that exchanges disulfides to dithiols and thus maintains protein functions under oxidative stress conditions [38, 39]. It also regenerates thiols of peroxiredoxins, the enzymes which reduce hydrogen peroxide, peroxynitrite, and organic hydroperoxides [40]. The disulfide groups of thioredoxin require reduction by thioredoxin reductases [41]. However, in the NC, thioredoxin reductase inhibiting factor (Txnip) [42] was found to be upregulated, pointing to inhibition of the thioredoxin system. Peroxiredoxins' expression was not induced in the NC, with the exception of Prdx4. Additionally, the sulfiredoxin 1 gene was upregulated. In the reaction with hydrogen peroxide, the thiols of peroxiredoxin IV undergo hyperoxidation to form cysteine sulfinic acid residues, which are subsequently reduced back to thiols in a two-step reaction catalyzed by sulfiredoxin 1 and thioredoxin/glutathione, respectively [43]. Hyperoxidized peroxiredoxins do not exert antioxidant activity but act as chaperons. They also enable transient local increase in hydrogen peroxide levels to favour the oxidation of signal transducing molecules, including apoptosis signal-regulating kinase 1, ASK1, which acts upstream of p38 MAPK and JNK [40]. ASK1 was inferred to participate in the signalling pathways operating in the NC [13]. Thus, peroxiredoxin 4 and sulfiredoxin 1 may constitute a node enabling propagation of oxidation-dependent signalling in the NC. The upregulation of other hydrogen peroxide-reducing enzymes GPX1, GPX3, and catalase, as well as SOD1 and SOD3, indicates that distinct redox networks are required for the precise regulation of ROS levels in various compartments of the NC. Although an uptake of physiological thiols to the NC through a collagen capsule cannot be excluded, it should be noted that their level in the NC may be limited due to a putative thioredoxin reductase inhibition and a lack of glutathione reductase upregulation. This reasoning conforms to the idea that depletion of reduced glutathione rather than antioxidant enzyme expression is a critical factor regulating ROS levels in senescence and during ageing [19, 44]. It should be emphasized that ROS exert a dual role in senescence; they may cause cellular damage that triggers senescence, and they may also act as second messengers in cellular signalling that controls senescence establishment [19]. In mouse models, both SOD1 deficiency and SOD3 overexpression were shown to be associated with increased cellular senescence and/or ageing [45–47]. Notably, the Gpx7 and Gpx8 genes were upregulated in the NC. These enzymes act as chaperones in the endoplasmic reticulum, but the reduction of their thiol groups relies on protein disulfide isomerase rather than glutathione [48].

Similar to p57 Kip2 and HO-1, antioxidant defence enzymes upregulated in the NC at the transcript and protein levels were localized not only to the NC but also to the larval cuticular region and internal organs, out of which the stichosome, whose canaliculi open to the esophagus, was well distinguished. The downregulation of SOD2 expression in the NC remains in accord with the NC-larva complex known to advance to anaerobic metabolism [49]. The nematode cuticle serves as an exoskeleton, extracellular matrix, and mechanical barrier whose permeability is regulated by collagens [50–52]. Adhesion of NC antioxidant enzymes to the cuticle would be the simplest way the larva can acquire antioxidant capacity. The other way could be absorption by the stichosome in the form of nutrients from the NC, both proteins and possibly RNAs. This function of the nematode digestive tract is commonly exploited in the free-living nematode *Caenorhabditis elegans*. RNA-mediated interference with the use of bacteria modified to express double stranded RNA is known as RNAi by feeding technique [53, 54]. Based on the antioxidant enzyme expression pattern identified in this study as parallel for the NC and larva, it can be hypothesized that the highly oxidative environment of the striated muscles [55, 56], even potentiated when regeneration is triggered due to the damage caused by larva penetration, favours larva settlement in this tissue. Cellular senescence is then induced in a portion of regenerating myofibre possibly in response to hypermitogenic signalling originating from myoblasts fusing with a regenerating myotube, with ROS acting as messengers of proliferative signal transduction [57–60]. Another reason for senescence initiation could be damage to macromolecules caused by excessive ROS production, which during muscle regeneration is kept under tight control, but in the condition of parasite infection, may be potentiated due to the immune response. Ultimate maintenance of NC senescence is proposed to be attributed to perpetual DNA damage caused by ROS generated by NADPH oxidase, whose expression may appear as a manifestation of immunological properties acquired by the NC in at least partly, AP-1-dependent manner [13]. Larva as a source of antigenic molecules may drive the NC towards antigen presentation functionality [13]. Antigen presentation ability is emerging as a general characteristic of senescent cells [61]. Finally, the larva takes advantage of NC senescence-specific gene expression to fulfil its needs for cell growth arrest and antioxidant capability. Accordingly, a high antioxidant capacity was shown to render muscle larvae resistant to the host immune response and to assure success in the next host colonization [62, 63]. It is a long-known phenomenon that *Trichinella* muscle larvae and adult forms are resistant to killing by oxidants, in contrast to newborn larvae, which are susceptible due to the lack of antioxidant enzymes [64]. To gain antioxidant capacity, newborn larvae should thus migrate to the environment of controlled oxidative stress, which is that of striated muscles. A high upregulation of antioxidant defence enzymes in the NC, out of which GPX3 and SOD3 show extracellular location [48, 65], well explains another long-known phenomenon of trichinellosis being accompanied by an increase in the antioxidant activities in the muscles and blood of infected animals [66–68].

The NC functionality is demonstrated in this study to be shaped by AP-1. Along with fibroblasts undergoing replicative senescence, transformed cells undergoing oncogene-induced senescence, and cancer cells driven into therapy-induced senescence [24–26, 61, 69], the NC transcriptome is also determined by AP-1. Consistent with AP-1, which is known to activate the expression of proinflammatory cytokines and chemokines and to control the immune response [70–72], numerous factors constituting SASP and regulating immune functions were upregulated in the NC. It can thus be hypothesized that AP-1 activated by oxidative stress is primarily involved in the regulation of NC immune properties.

## 5. Conclusions

We showed in the current study that cellular senescence of the NC harbouring *Trichinella* spp. muscle larvae was underpinned by oxidative stress, the upregulation of antioxidant defence enzymes, and the activation of the AP-1 transcription factor. Growth arrest-associated factors, as well as antioxidant enzymes, were upregulated in the NC at the transcript and protein levels, and they were also present in the larva. Therefore, it is hypothesized that larva took advantage of the NC proteins to fulfil its needs for growth arrest and antioxidant capacity. The NC is proposed to be established in the course of muscle regeneration influenced by parasite antigenic properties. Cellular senescence apparently represents the state of metabolism that constitutes a niche suitable for larva long-term existence in the muscles. This finding opens up the possibility for a new therapeutic approach to trichinellosis with the use of senolytic drugs that selectively eliminate senescent cells in rodents and humans [73]. As senolytics were demonstrated to alleviate symptoms of many age-related dysfunctions and even to prolong the life span in rodents [74], several clinical trials for drug candidates that systemically decrease senescent cell burden are currently ongoing [75].

## Data Availability

The data that support the findings of this study are openly available in ArrayExpress repository at https://www.ebi.ac.uk/arrayexpress, with E-MTAB-12042 reference number.

## Supplementary Materials

Supplementary Materials Supplementary Figure 1: nurse cell-*Trichinella spiralis* muscle larva complexes within skeletal muscle. Supplementary Figure 2: representative preparation of NC-larva complexes isolated from murine muscles by enzymatic digestion. Supplementary Figure 3: SA-*β*-Gal activity assayed in a NC isolated by enzymatic digestion. Supplementary Table 1: expression of antioxidant defence enzymes in the NC, as analyzed by transcriptomic approaches. Supplementary Table 2: AP-1 target genes whose expression was induced in the NC in comparison to C2C12 myoblasts and myotubes, as analyzed by competitive expression microarrays.
