# Supplementary material for: Novel Role of Mammalian Cell Senescence-Sustenance of Muscle Larvae of Trichinella spp
Source: Oxid Med Cell Longev. 2022 Nov 28;2022:1799839. doi: 10.1155/2022/1799839 (PMC9722307; doi:10.1155/2022/1799839)
Supplement: Supplementary Materials — Supplementary Figure 1: nurse cell-Trichinella spiralis muscle larva complexes within skeletal muscle. Supplementary Figure 2: representative preparation of NC-larva complexes isolated from murine muscles by enzymatic digestion. Supplementary Figure 3: SA-β-Gal activity assayed in a NC isolated by enzymatic digestion. Supplementary Table 1: expression of antioxidant defence enzymes in the NC, as analyzed by transcriptomic approaches. Supplementary Table 2: AP-1 target genes whose expression was induced in the NC in comparison to C2C12 myoblasts and myotubes, as analyzed by competitive expression microarrays. [file 1799839.f1.docx]

Supplementary Figure 1: Nurse Cell-*Trichinella spiralis* muscle larva complexes within skeletal muscle. A fragment of muscle isolated from an infected mouse was compressed and imaged using an Opta-Tech MW50 inverted microscope (Opta-Tech, Warsaw, Poland), at 100x magnification. Spirally twisted larvae within the NCs are visible. A single NC containing two larvae is marked with a drumstick. Asterisks indicate representative regions of the NC cytoplasm. Arrows point to collagen capsule where the capsule is best distinguishable.


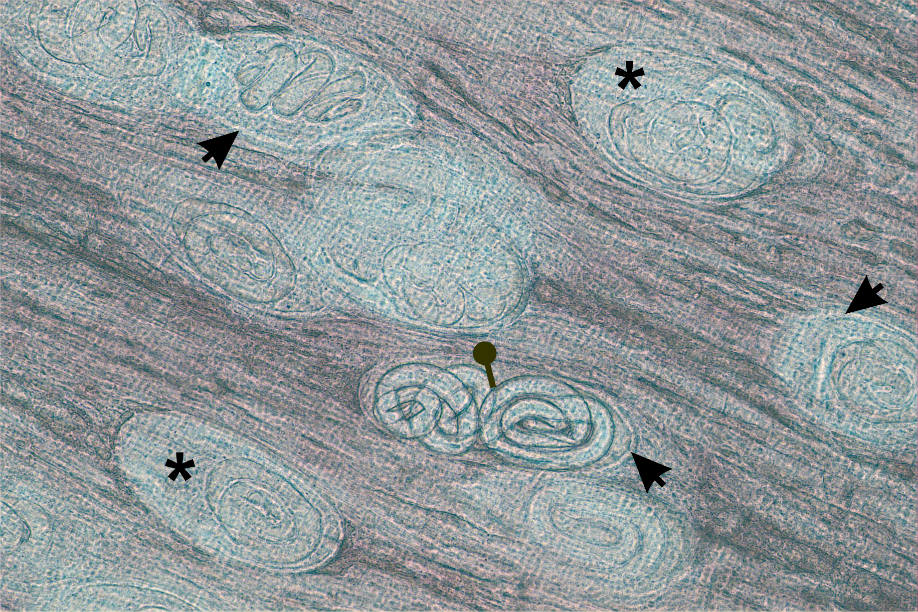


Supplementary Figure 2: Representative preparation of NC-larva complexes isolated from murine muscles by digestion with 0.25% pronase, 0.1% collagenase and 0.1% hyaluronidase [12]. Representative NC areas consisting of the cytoplasm and collagen capsule are marked with asterisks. The image was taken with a Nikon Optiphot-Z microscope at 100x magnification.


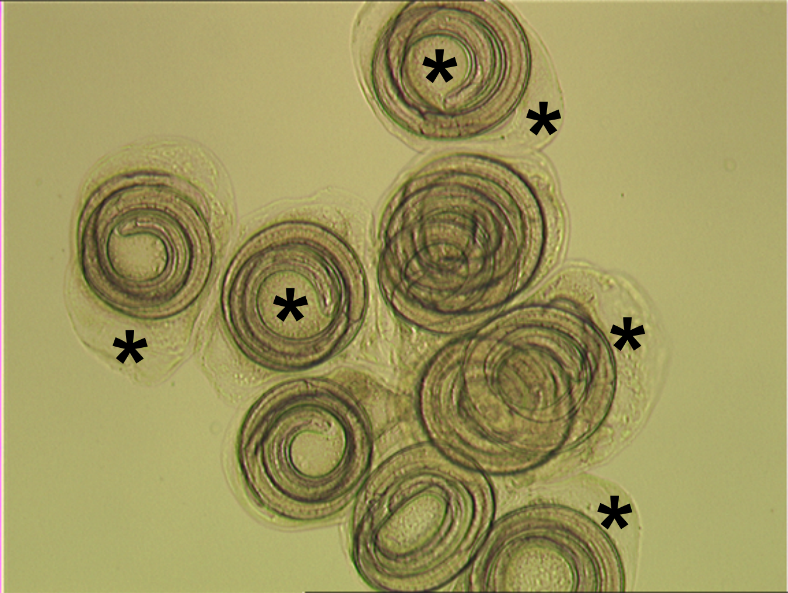


Supplementary Figure 3: SA-β-Gal activity assayed in a NC isolated by enzymatic digestion, as described under Materials and Methods. Blue-stained NC cytoplasm areas are marked by asterisks. Collagen capsule is indicated by the arrows.


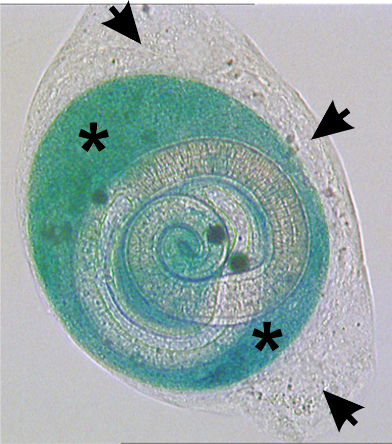


Supplementary Table 1: Expression of antioxidant defence enzymes in the NC, as analyzed by transcriptomic approaches described under Materials and Methods. PCR Array data are given as 2exp-ΔC_T_ where ΔC_T_ is C_T_ (target)-C_T_ (Gapdh), followed by average deviation in parentheses. Microarray data are given as log_2_FC in gene expression in the NC in comparison to C2C12 myoblasts and myotubes, followed by *p*-value in parentheses.

| GenBank | Gene symbol | Description | Gene expression | |
| --- | --- | --- | --- | --- |
|  |  |  | 2^-ΔCT^ (±AD, *n*=2) | log_2_FC  (*p*-value) in NC *vs.* C2C12 myoblasts/  myotubes |
| **Oxidative stress markers** | | | | |
| NM_010442 | Hmox1 | Heme oxygenase (decycling) 1 |  | 1.45 (0.00) /  2.07 (0.00) |
| NM_010479 | Hsp1a | Heat shock protein 1A |  | 4.91 (0.00) /  4.51 (0.00) |
| **Glutathione antioxidant defense system** | | | | |
| NM_008160 | Gpx1 | Glutathione peroxidase 1 | 22.97 (±2.42) | / 1.03 (0.00) |
| NM_030677 | Gpx2 | Glutathione peroxidase 2 | 0.0003 (±0.00025) |  |
| NM_008161 | Gpx3 | Glutathione peroxidase 3 | 13.64 (±0.99) | 3.44 (0.00) / 2.50 (0.00) |
| NM_008162 | Gpx4 | Glutathione peroxidase 4 | 1.41 (±0.20) |  |
| NM_010343 | Gpx5 | Glutathione peroxidase 5 | 4.24E-05 (±1.15E-05) |  |
| NM_145451 | Gpx6 | Glutathione peroxidase 6 | 0.0007 (±0.0001) |  |
| NM_024198 | Gpx7 | Glutathione peroxidase 7 | 0.0015 (±7.43E-05) | 3.96 (0.00) /  3.73 (0.00) |
| NM_027127 | Gpx8 | Glutathione peroxidase 8 | 10.73 (±0.31) | 1.20 (0.01) /  1.65 (0.00) |
| NM_010344 | Gsr | Glutathione reductase | 0.36 (±0.068) | -0.93 (0.01) / |
| **Thioredoxin antioxidant defense system** | | | | |
| NM_011034 | Prdx1 | Peroxiredoxin 1 | 1.23 (±0.03) |  |
| NM_011563 | Prdx2 | Peroxiredoxin 2 | 0.19 (±0.013) | -0.81 (0.01) /  -0.73 (0.02) |
| NM_007452 | Prdx3 | Peroxiredoxin 3 | 1.48 (±0.28) | -1.05 (0.00) /  -1.21 (0.00) |
| NM_016764 | Prdx4 | Peroxiredoxin 4 | 11.26 (±1.15) | / 1.79 (0.00) |
| NM_012021 | Prdx5 | Peroxiredoxin 5 | 2.54 (±0.31) |  |
| NM_007453 | Prdx6 | Peroxiredoxin 6 | 0.34 (±0.023) | -1.51 (0.00) /  -0.99 (0.00) |
| NM_029688 | Srxn1 | Sulfiredoxin 1 homolog (S. cerevisiae) | 0.60 (±0.047) | 0.72 (0.05) /  2.85 (0.00) |
| NM_015762 | Txnrd1 | Thioredoxin reductase 1 | 1.94 (±0.12) | / 1.54 (0.00) |
| NM_013711 | Txnrd2 | Thioredoxin reductase 2 | 0.072 (±0.0149) |  |
| NM_153162 | Txnrd3 | Thioredoxin reductase 3 | 0.40 (±0.029) |  |
| NM_023719 | Txnip | Thioredoxin interacting protein | 3.56 (±0.39) | 0.94 (0.01) /  0.77 (0.00) |
| **Catalase and superoxide dismutase molecules** | | | | |
| NM_009804 | Cat | Catalase | 4.57 (±0.11) | 0.53 (0.02) /  0.48 (0.01) |
| NM_011434 | Sod1 | Superoxide dismutase 1, soluble | 17.12 (±1.72) | / 1.31 (0.00) |
| NM_013671 | Sod2 | Superoxide dismutase 2, mitochondrial | 0.14 (±0.01) | /-1.11 (0.00) |
| NM_011435 | Sod3 | Superoxide dismutase 3, extracellular | 1.09 (±0.09) | 2.05 (0.00) /  1.42 (0.00) |
| NM_016892 | Ccs | Copper chaperone for superoxide dismutase | 0.40 (±0.13) | 0.76 (0.00) /  0.54 (0.01) |

Supplementary Table 2: AP-1 target genes whose expression was induced in the NC in comparison to C2C12 myoblasts and myotubes, as analyzed by competitive expression microarrays described under Materials and Methods. Genes were identified as AP-1 targets by referral of microarray datasets to the canonical AP-1 target gene lists available at <http://rulai.cshl.edu/TRED/GRN/AP1.htm>, as well as in the references [15,76-80]. Gene expression is given as log_2_FC followed by *p*-value in parentheses with the parameter cut off values set at -0.5≥log_2_FoldChange (FC) ≥0.5 and *p*-value≤0.05. Gene symbols printed in capital letters indicate that gene was extracted either from canonical human AP-1 target gene list or from both human and murine AP-1 target gene lists. Gene symbols which start with a capital letter indicate that gene was extracted from murine AP-1 target gene list. The lack of figure in the expression column indicates that gene expression was not changed *vs.* either C2C12 myoblasts or myotubes.

| GenBank | Gene symbol | Description | Gene expression  log_2_FC  (*p*-value) in NC *vs.* C2C12 myoblasts / myotubes |
| --- | --- | --- | --- |
| NM_013454 | ABCA1 | ATP-binding cassette, sub-family A (ABC1), member 1 | 4.85 (0.00) / 1.65 (0.02) |
| NM_011075 | ABCB1 | ATP-binding cassette, sub-family B (MDR/TAP), member 1B | / 1.06 (0.00) |
| NM_007388 | Acp5 | Acid phosphatase 5, tartrate resistant | 1.82 (0.03) / 1.45 (0.00) |
| NM_019811 | ACSS2 | Acyl-CoA synthetase short-chain family member 2 | 1.49 (0.01) / |
| NM_009696 | APOE | Apolipoprotein E | 4.58 (0.00) / 4.25 (0.00) |
| NM_007471 | APP | Amyloid beta (A4) precursor protein | 2.94 (0.00) / 1.87 (0.00) |
| NM_007498 | ATF3 | Activating transcription factor 3 | 3.40 (0.00) / 3.23 (0.00) |
| NM_009738 | BCHE | Butyrylcholinesterase | 1.29 (0.02) / |
| NM_031161 | CCK | Cholecystokinin | 2.50 (0.02)/ 1.88 (0.01) |
| NM_011333 | CCL2 | Chemokine (C-C motif) ligand 2 | / 1.23 (0.01) |
| NM_013652 | CCL4 | Chemokine (C-C motif) ligand 4 | 6.59 (.00) / 6.46 (0.00) |
| NM_013653 | CCL5 | Chemokine (C-C motif) ligand 5 | 4.02 (0.00) / 2.83 (0.00) |
| NM_013654 | CCL7 | Chemokine (C-C motif) ligand 7 | / 1.09 (0.00) |
| NM_007631 | CCND1 | Cyclin D1 | / 3.24 (0.00) |
| NM_009829 | CCND2 | Cyclin D2 | 2.62 (0.00) / 3.46 (0.00) |
| NM_009917 | CCR5 | Chemokine (C-C motif) receptor 5 | 3.36 (0.01) / 2.63 (0.00) |
| NM_007719 | CCR7 | Chemokine (C-C motif) receptor 7 | 1.54 (0.03) / 1.14 (0.01) |
| NM_007669 | CDKN1A | Cyclin-dependent kinase inhibitor 1A (P21) | 1.22 (0.02) / -2.02 (0.00) |
| NM_009877 | Cdkn2a | Cyclin-dependent kinase inhibitor 2A (Cdkn2a), transcript variant 1, p16 | 3.66 (0.00) / 3.46 (0.00) |
| NM_009851 | CD44 | CD44 antigen | / 1.61 (0.00) |
| NM_001033122 | CD69 | CD69 antigen | 1.94 (0.05) / 1.19 (0.03) |
| NM_009883 | Cebpb | CCAAT/enhancer binding protein (C/EBP), beta | 1.06 (0.04) / 0.80 (0.01) |
| NM_013492 | CLU | Clusterin | 4.65 (0.00) / 4.49 (0.00) |
| NM_007742 | COL1A1 | Procollagen, type I, alpha 1 | 2.80 (0.00) / 2.44 (0.00) |
| NM_007743 | COL1A2 | Procollagen, type I, alpha 2 | 1.14 (0.01) / 1.58 (0.00) |
| NM_015734 | Col5a1 | Procollagen, type V, alpha 1 | 1.09 (0.05) / |
| NM_007779 | CSF1R | Colony stimulating factor 1 receptor, transcript variant 2 | 3.61 (0.00) / 3.49 (0.00) |
| NM_007793 | CSTB | Cystatin B | 0.71 (0.00) / 1.46 (0.00) |
| NM_009984 | CTSL | Cathepsin L | 1.45 (0.00) / 0.86 (0.01) |
| NM_009140 | Cxcl2 | Chemokine (C-X-C motif) ligand 2 | 4.97 (0.01) / 4.24 (0.00) |
| NM_007806 | CYBA | Cytochrome b-245, alpha polypeptide | 3.90 (0.00) / 3.80 (0.00) |
| NM_007833 | DCN | Decorin | 3.69 (0.00) / -1.11 (0.00) |
| NM_007868 | DMD | Dystrophin, muscular dystrophy | 2.88 (0.00) / |
| NM_170778 | DPYD | Dihydropyrimidine dehydrogenase | 3.77 (0.00) / 2.88 (0.00) |
| NM_007899 | Ecm1 | Extracellular matrix protein 1 | 1.58 (0.00) / 2.09 (0.00) |
| NM_007912 | Egfr | Epidermal growth factor receptor, transcript variant 2 | 2.18 (0.00) / 1.20 (0.00) |
| NM_007913 | EGR1 | Early growth response 1 | 1.34 (0.00) / |
| NM_133918 | EMILIN1 | Elastin microfibril interfacer 1 | 2.25 (0.00) / 2.62 (0.00) |
| NM_007956 | ESR1 | Estrogen receptor 1 (alpha) | 2.10 (0.02) / |
| NM_011808 | ETS1 | Mus musculus E26 avian leukemia oncogene 1, 5' domain (Ets1), transcript variant 1 | 0.72 (0.02) / 1.18 (0.00) |
| NM_177333 | EXOC3 | Exocyst complex component 3 | 2.24 (0.00) / 0.55 (0.01) |
| NM_010171 | F3 | Coagulation factor III | 0.84 (0.03) / 2.88 (0.00) |
| NM_024406 | Fabp4 | Fatty acid binding protein 4, adipocyte | 2.30 (0.00) / 3.31 (0.01) |
| NM_007987 | FAS | Fas (TNF receptor superfamily member) | 2.58 (0.00) / 1.10 (0.02) |
| NM_010206 | Fgfr1 | Fibroblast growth factor receptor 1 | 1.26 (0.01) / 1.20 (0.01) |
| NM_008013 | FGL2 | Fibrinogen-like protein 2 | 3.05 (0.00) / 3.84 (0.00) |
| NM_008036 | FOSB | FBJ osteosarcoma oncogene B | 2.59 (0.00) / 2.47 (0.00) |
| NM_010235 | FOSL1 | Fos-like antigen 1 | -1.62 (0.00) / 1.04 (0.00) |
| NM_008077 | Gad1 | Glutamic acid decarboxylase 1 | 0.64 (0.02) / |
| NM_008094 | GBA | Glucosidase, beta, acid | 1.20 (0.01) / 1.15 (0.00) |
| NM_010358 | Gstm1 | Glutathione S-transferase, mu 1 | 0.91 (0.01) / 1.32 (0.00) |
| NM_010415 | HBEGF | Heparin-binding EGF-like growth factor | 2.65 (0.00) / 3.13 (0.00) |
| NM_010422 | HEXB | Hexosaminidase B | 2.16 (0.01) / 2.55 (0.00) |
| NM_010424 | HFE | Hemochromatosis | 2.19 (0.00) / 1.90 (0.01) |
| NM_010391 | HLA-A (H2-Q10) | Mus musculus histocompatibility 2, Q region locus 10 (H2-Q10) | 1.74 (0.02) / 1.72 (0.01) |
| NM_010380 | HLA-C (H2-D1) | Mus musculus histocompatibility 2, D region locus 1 (H2-D1) | 1.98 (0.02) / 1.82 (0.02) |
| NM_010378 | HLA-DQA1 (H2-Aa) | Mus musculus histocompatibility 2, class II antigen A, alpha (H2-Aa) | 3.65 (0.02) / 3.60 (0.01) |
| NM_010381 | HLA-DRA (H2-Ea) | Mus musculus histocompatibility 2, class II antigen E alpha (H2-Ea) | 3.24 (0.02) / 2.50 (0.01) |
| NM_013551 | HMBS | Hydroxymethylbilane synthase | / 1.01 (0.00) |
| NM_010442 | HMOX1 | Heme oxygenase (decycling) 1 | 1.45 (0.00) / 2.07 (0.00) |
| NM_008285 | HRH1 | Histamine receptor H 1 | / 1.21 (0.01) |
| NM_010479 | HSPA1A | Heat shock protein 1A | 4.92 (0.00) / 4.51 (0.00) |
| NM_010493 | ICAM1 | Intercellular adhesion molecule | 1.75 (0.03) / 1.10 (0.01) |
| NM_010500 | IER5 | Immediate early response 5 | 1.30 (0.01) / 1.88 (0.01) |
| NM_010510 | IFNB1 | Interferon beta 1, fibroblast | / 1.98 (0.05) |
| NM_008337 | IFNG | Interferon gamma | 2.94 (0.02) / 1.67 (0.03) |
| NM_184052 | IGF1 | Insulin-like growth factor 1, transcript variant 2 | 2.64 (0.00) / 0.54 (0.01) |
| NM_008342 | IGFBP2 | Insulin-like growth factor binding protein 2 | -1.84 (0.01) / 1.92 (0.02) |
| NM_010517 | IGFBP4 | Insulin-like growth factor binding protein 4 | 2.97 (0.00) / 2.70 (0.00) |
| NM_010591 | JUN | Jun oncogene | 0.94 (0.00) / |
| NM_010554 | IL1A | Interleukin 1 alpha | 2.74 (0.01) / 2.32 (0.00) |
| NM_008361 | IL1B | Interleukin 1 beta | 6.29 (0.00) / 4.68 (0.00) |
| NM_008352 | IL12B | Interleukin 12b | 1.88 (0.01) / 1.78 (0.05) |
| NM_008368 | IL2RB | Interleukin 2 receptor, beta chain | 1.53 (0.02) / |
| NM_031168 | IL6 | Interleukin 6 | 1.85 (0.02) / |
| NM_021334 | ITGAX | Integrin alpha X | 2.99 (0.00) / 2.70 (0.00) |
| NM_010637 | Klf4 | Kruppel-like factor 4 (gut) | 1.82 (0.00) / 1.10 (0.00) |
| NM_010664 | KRT18 | Keratin complex 1, acidic, gene 18 | 0.62 (0.05) / 0.70 (0.02) |
| NM_008489 | LBP | Lipopolysaccharide binding protein | 2.22 (0.00) / 1.59 (0.00) |
| NM_008879 | LCP1 | Lymphocyte cytosolic protein 1 | 4.04 (0.00) / 4.23 (0.00) |
| NM_008508 | LOR | Loricrin | 1.74 (0.01) / 2.08 (0.01) |
| NM_172778 | MAOB | Monoamine oxidase B | 3.86 (0.00) / 3.63 (0.00) |
| NM_011952 | Mapk3 | Mitogen activated protein kinase 3, Erk1 | 0.77 (0.00) / |
| NM_008597 | MGP | Matrix Gla protein | 1.51 (0.01) / 1.30 (0.01) |
| NM_008605 | MMP12 | Matrix metallopeptidase 12 | 1.38 (0.01) / 1.11 (0.03) |
| NM_008607 | MMP13 | Matrix metallopeptidase 13 | 2.31 (0.02) / 1.73 (0.00) |
| NM_021412 | MMP19 | Matrix metallopeptidase 19 | 1.53 (0.00) / 1.21 (0.04) |
| NM_008610 | MMP2 | Matrix metallopeptidase 2 | 2.60 (0.00) / 0.92 (0.00) |
| NM_010809 | MMP3 | Matrix metallopeptidase 3 | 5.92 (0.00) / 4.99 (0.00) |
| NM_031195 | MSR1 | Macrophage scavenger receptor 1 | 2.65 (0.00) / 2.20 (0.00) |
| NM_013603 | Mt3 | Metallothionein 3 | / 0.60 (0.01) |
| NM_010840 | MTHFR | 5,10-methylenetetrahydrofolate reductase | 0.79 (0.00) / |
| NM_008673 | NAT1 | N-acetyltransferase 1 (arylamine N-acetyltransferase) | 1.44 (0.05) / 0.93 (0.03) |
| NM_010877 | NCF2 | Neutrophil cytosolic factor 2 | 3.15 (0.01) / 3.023 (0.00) |
| NM_016791 | Nfatc1 | Nuclear factor of activated T-cells, cytoplasmic, calcineurin-dependent 1, transcript variant 1 | 1.00 (0.03) / 0.98 (0.01) |
| NM_008697 | NIN | Ninein | / 1.09 (0.01) |
| NM_008725 | NPPA | Natriuretic peptide precursor type A | 0.97 (0.04) / |
| NM_023456 | NPY | Neuropeptide Y | 1.63 (0.01) / 1.30 (0.00) |
| NM_008706 | NQO1 | NAD(P)H dehydrogenase, quinone 1 | -1.76 (0.00) / 1.02 (0.01) |
| NM_008173 | Nr3c1 | Nuclear receptor subfamily 3, group C, member 1 | 0.93 (0.02) / 0.86 (0.00) |
| NM_013613 | Nr4a2 | Nuclear receptor subfamily 4, group A, member 2 | / 1.13 (0.02) |
| NM_145227 | OAS2 | 2'-5' oligoadenylate synthetase 2 | 0.55 (0.01) / |
| NM_008777 | Pah | Phenylalanine hydroxylase | 0.73 (0.04) / |
| NM_001002927 | PENK (Penk1) | Preproenkephalin 1 | 3.00 (0.00) / 3.22 (0.00) |
| NM_008872 | PLAT | Plasminogen activator, tissue | 2.46 (0.00) / 2.77 (0.00) |
| NM_008873 | PLAU | Plasminogen activator, urokinase | / 1.44 (0.03) |
| NM_011113 | PLAUR | Plasminogen activator, urokinase receptor | -1.23 (0.02) / 2.50 (0.00) |
| NM_011144 | PPARA | Peroxisome proliferator activated receptor alpha | 1.98 (0.00) / 1.57 (0.00) |
| NM_008923 | Prkar1b | Protein kinase, cAMP dependent regulatory, type I beta | 2.92 (0.00) / 2.68 (0.00) |
| NM_008855 | PRKCB1 | Protein kinase C, beta 1 | 3.33 (0.01) / 2.75 (0.00) |
| NM_011170 | PRNP | Prion protein | 3.19 (0.00) / 1.56 (0.00) |
| NM_011179 | PSAP | Prosaposin | 1.67 (0.00) / 1.15 (0.00) |
| NM_011255 | RBP4 | Retinol binding protein 4, plasma | 5.05 (0.00) / 4.90 (0.00) |
| NM_011519 | Sdc1 | Syndecan 1 | / 0.63 (0.03) |
| NM_011521 | Sdc4 | Syndecan 4 | 0.72 (0.00) / 0.81 (0.01) |
| NM_027016 | SEC62 (Tloc1) | Translocation protein 1 | 1.28 (0.00) / 0.85 (0.00) |
| NM_008871 | SERPINE1 | Serine (or cysteine) peptidase inhibitor, clade E, member 1 | 2.47 (0.01) / 3.92 (0.00) |
| NM_011111 | SERPINB2 | Serine (or cysteine) peptidase inhibitor, clade B, member 2 | -2.60 (0.01) / 1.00 (0.04) |
| NM_009160 | SFTPD | Surfactant associated protein D | / 0.50 (0.01) |
| NM_015747 | Slc20a1 | Solute carrier family 20, member 1 | / 1.76 (00.01) |
| NM_008577 | SLC3A2 | Solute carrier family 3 (activators of dibasic and neutral amino acid transport), member 2 | 2.76 (0.00) / 2.73 (0.00) |
| NM_010484 | SLC6A4 | Solute carrier family 6 (neurotransmitter transporter, serotonin), member 4 | 0.78 (0.02) / 0.85 (0.01) |
| NM_009320 | SLC6A6 | Solute carrier family 6 (neurotransmitter transporter, taurine), member 6 | 2.04 (0.00) / |
| NM_008540 | SMAD4 | MAD homolog 4 (Drosophila) | 1.27 (0.01) / 1.28 (0.00) |
| NM_008543 | SMAD7 | MAD homolog 7 (Drosophila) | 1.58 (0.04) / 2.18 (0.01) |
| NM_011434 | SOD1 | Superoxide dismutase 1, soluble | / 1.31 (0.00) |
| NM_011355 | SPI1 | Mus musculus SFFV proviral integration 1 (Sfpi1) | 0.58 (0.03) / |
| NM_013761 | SRR | Serine racemase | 0.88 (0.04) / |
| NM_009183 | St8sia4 | ST8 alpha-N-acetyl-neuraminide alpha-2,8-sialyltransferase 4 | 4.04 (0.00) /3.57 (0.01) |
| NM_001025313 | Tapbp | TAP binding protein, transcript variant 1 | 1.14 (0.01) / 0.56 (0.01) |
| NM_011539 | TBXAS1 | Thromboxane A synthase 1, platelet | 2.54 (0.01) / 2.52 (0.00) |
| NM_011571 | Tesk1 | Testis specific protein kinase 1 | 0.72 (0.03) / |
| NM_009364 | TFPI2 | Tissue factor pathway inhibitor 2 | 1.48 (0.01) / |
| NM_011577 | TGFB1 | Transforming growth factor, beta 1 | 0.59 (0.02) / |
| NM_009367 | TGFB2 | Transforming growth factor, beta 2 | 2.20 (0.00) / 1.67 (0.00) |
| NM_011594 | TIMP2 | Tissue inhibitor of metalloproteinase 2 | 3.06 (0.00) / 1.49 (0.00) |
| NM_011595 | Timp3 | Tissue inhibitor of metalloproteinase 3 | 2.05 (0.04) / 2.08 (0.02) |
| NM_021297 | Tlr4 | Toll-like receptor 4 | 1.60 (0.03) / |
| NM_031178 | TLR9 | Toll-like receptor 9 | 0.69 (0.00) / |
| NM_011607 | TNC | Tenascin C | / 1.60 (0.00) |
| NM_013693 | TNF | Tumor necrosis factor | 3.13 (0.00) / 2.85 (0.00) |
| NM_009398 | TNFAIP6 | Tumor necrosis factor alpha induced protein 6 | 3.81 (0.01) / 3.10 (0.00) |
| NM_020275 | TNFRSF10B | Tumor necrosis factor receptor superfamily, member 10b | 1.66 (0.00) / 1.63 (0.00) |
| NM_008764 | Tnfrsf11b | Tumor necrosis factor receptor superfamily, member 11b (osteoprotegerin) | 4.69 (0.00) / 3.89 (0.00) |
| NM_011691 | VAV1 | vav 1 oncogene | 2.43 (0.00) / 2.20 (0.00) |
| NM_011701 | Vim | Vimentin | -0.87 (0.02) / 0.75 (0.03) |
